# Combined sterile insect technique and incompatible insect technique: sex separation and quality of sterile *Aedes aegypti* male mosquitoes released in a pilot population suppression trial in Thailand

**DOI:** 10.1186/s13071-018-3214-9

**Published:** 2018-12-24

**Authors:** Patttamaporn Kittayapong, Nuanla-ong Kaeothaisong, Suwannapa Ninphanomchai, Wanitch Limohpasmanee

**Affiliations:** 1 Center of Excellence for Vectors and Vector-Borne Diseases, Faculty of Science, Mahidol University at Salaya, Nakhon Pathom, 73170 Thailand; 2 Department of Biology, Faculty of Science, Mahidol University, Bangkok, 10400 Thailand; 3 Thailand Institute of Nuclear Technology, Ministry of Science and Technology, Nakhon Nayok, 26120 Thailand

**Keywords:** Mosquito vector, Pupal size, SIT, Sterility, Longevity, *Wolbachia*, IIT

## Abstract

**Background:**

The sterile insect technique (SIT), which is based on irradiation-induced sterility, and incompatible insect technique (IIT), which is based on *Wolbachia*-induced cytoplasmic incompatibility (a kind of male sterility), have been used as alternative methods to reduce mosquito vector populations. Both methods require the release of males to reduce fertile females and suppress the number of natural populations. Different techniques of sex separation to obtain only males have been investigated previously. Our work involves an application of mechanical larval-pupal glass separators to separate *Wolbachia*-infected *Aedes aegypti* males from females at the pupal stage, prior to irradiation, and for use in a pilot field release and to assess the quality of males and females before and after sex separation and sterilization.

**Results:**

This study was the first to demonstrate the efficiency of mechanical glass separators in separating males for use in an *Ae. aegypti* suppression trial by a combined SIT/IIT approach. Our results indicated that male and female pupae of *Wolbachia*-infected *Ae. aegypti* mosquitoes were significantly different (*p* < 0.05) in weight, size, and emergence-time, which made it easier for sex separation by this mechanical method. During the pilot field release, the percentage of female contamination was detected to be quite low and significantly different between the first (0.10 ± 0.13) and the second (0.02 ± 0.02) twelve-week period. Both males and females were almost completely sterile after exposure to 70 Gy irradiation dose. We observed that both irradiated *Wolbachia*-infected males and females survived and lived longer than two weeks, but males could live longer than females (*p* < 0.05) when they were irradiated at the same irradiation dose. When comparing irradiated mosquitoes with non-irradiated ones, there was no significant difference in longevity and survival-rate between those males, but non-irradiated females lived longer than irradiated ones (*p* < 0.05).

**Conclusion:**

Mechanical sex separation by using a larval-pupal glass separator was practically applied to obtain only males for further sterilization and open field release in a pilot population suppression trial of *Ae. aegypti* in Thailand. Female contamination was detected to be quite low, and skilled personnel can reduce the risk for female release. The irradiated *Wolbachia*-infected females accidentally released were found to be completely sterile, with shorter life span than males.

## Introduction

Dengue, chikungunya and Zika virus infections are mosquito-borne diseases that pose major public health problems in many countries where *Aedes aegypti* are dominant mosquito vectors. Since traditional vector control strategies do not provide satisfactory results, alternative eco-friendly techniques have been proposed to control mosquito vectors in many countries, including the sterile insect technique (SIT) as a component of an area-wide integrated vector management (AW-IVM) programme [1].

Another approach in sterilizing males is to exploit the phenomenon of *Wolbachia*-induced cytoplasmic incompatibility (CI), which is expressed as embryonic lethality induced through mating between *Wolbachia*-infected males and uninfected females, or females infected with different *Wolbachia* strains [1, 2]. Recently, a combined sterile insect technique and incompatible insect technique (SIT/IIT) has been proposed as a means to introduce sterility into target populations of insect pests and disease vectors, including mosquitoes [3, 4]. In general, *Wolbachia*-infected females could be sterilized with a minimum dose of radiation that leads to complete sterility. As a result, any accidentally released females should be sterile and this would eliminate the risk of replacement of natural populations with *Wolbachia*-infected ones [1, 5–10].

Population suppression using the combined SIT/IIT approach requires release of a large number of male mosquitoes; therefore, an efficient separation between males and females is essential in order to produce and release only sterile males into the environment. Many studies have attempted to develop sex separation methods, based on biological, genetic and transgenic approaches, in order to support the application of SIT in mosquito control. Sieving technique was introduced by taking into account size difference between male and female pupae [11, 12]. The development of genetic sexing strains (GSS) as well as other sex separation strategies are currently under development and/or refinement but none of them have so far succeeded to eliminate the females in order to achieve male only releases for SIT or other related applications [13–15].

Sterility in wild populations could be introduced on the condition that sterile male mosquitoes are of good quality and are able to compete with wild males. Previous studies have reported that the effect of irradiation and/or *Wolbachia* infection is minimal, if any, on the biological quality of *Aedes albopictus* by assessing traits such as egg-hatching rate, survival of pupae and adults, sex ratio, duration of larval stages, time to adult emergence, wing length, female fecundity, longevity, sterility and mating competitiveness [8, 16, 17]. In this study, and in the frame of a combined SIT/IIT population suppression trial in Thailand, we report the quality of *Wolbachia*-infected *Ae. aegypti* male and female pupae in term of differences in weight, size and developmental-time following sex separation. The quality of irradiated emerged males, i.e. sterility, survival and longevity that were important for the success of the suppression trial, was also investigated.

## Methods

### Rearing of *Aedes aegypti* mosquito colony

*Aedes aegypti* mosquitoes, used in the present study, were originally collected from villages in Pleang Yao District, Chachoengsao Province, while *Aedes albopictus* were collected from rubber plantations from the same area. A *Wolbachia-*transinfected *Ae. aegypti* colony was obtained from direct microinjection using the *Aedes aegypti* mosquito colony and *Wolbachia* strains from the *Ae. albopictus* collected from the same origin as those reported in Ruang-areerate and Kittayapong (2006) [18]. The establishment and characteristics of this *Wolbachia*-infected mosquito strain were demonstrated in Ruang-areerate and Kittayapong (2006) [18].

In these experiments, mosquitoes were reared in an aluminum cage sized 30 cm x 30 cm x 30 cm, fed with 10 % sucrose solution with 75 ± 2 % humidity, 27 ± 2 °C, at a photoperiod of L12:D12 in a screen climatic control insectary at the Center of Excellence for Vectors and Vector-Borne Diseases, Faculty of Science, Mahidol University at Salaya, Nakhon Pathom, Thailand. Female mosquitoes were fed with pig blood, obtained from a qualified slaughterhouse, for 3-4 consecutive days after mating. The Hemotek blood-feeding system (Hemotek Ltd., UK), containing 20 ml of pig blood, was placed on top of the cage for 1-2 hours of each feeding cycle. Egg papers were placed in the containers inside the cage following blood-feeding. After 3-4 days, the egg papers were then collected, dried and transferred into glass containers with screw-top covers filled with deionized water for egg hatching. After the eggs were hatched into the first-instar larvae, they were counted and transferred into plastic trays sized 32 cm × 42 cm × 5 cm, each containing about 2000 larvae. After egg hatching, larval diet was provided daily, at a total quantity of 6.5 g. The larval diet composed of mixed fish meal (Chanpongcharoen Kankaset Supplier, Thailand), pork liver powder and yeast (*Saccharomyces cerevisiae*) (Cheese Powder Supplier, Thailand) at a ratio of 5:4:1 respectively. No larval diet was given when larvae reached the pupal stage, which took about 6-7 days. Pupae were then placed in plastic containers prior to sex separation.

### Mechanical separation of male and female pupae

Pupae were separated into different layers of males and females using larval-pupal glass separators (Model 5412, John W. Hock Company, Gainesville, FL, USA). Each unit consisted of horizontal aluminum plates supporting two glass panels that formed between them an adjustable, downward-pointing, wedge-shaped space into which the pupae could be filled. The numbers of pupae were thus separated on the basis of size by regulating the thickness and angle of the wedge-shaped space by means of four control knobs in each of the four angles. The lower opening was adjusted so that the larger female pupae were retained in a layer in the tapering space between the panels of glasses. The smaller male pupae were drained through into a receiving container placed below. The operation was completed by opening the wedge and flushing the female pupae into a second receiving container.

For each sex separation, one liter of water that contained about 1500 to 2000 mixed male and female pupae were introduced into the system. Adjustment of the glass panels was performed gradually, and water circulation was supplied all along the process in order to push and wash the pupae down into the container. The pupae maintained between the plates was varied by adjusting the angle of the plates. The smaller male pupae were flushed out first and then collected in plastic containers, whereas the bigger female pupae were collected after, and the cycle continued. One cycle of 1500-2000 pupae varied on average between 2-5 minutes, but it could take a longer time if there was a mixture of larvae inside. After counting, the male pupae were transferred into a plastic cup for further transportation to the radiation source. The process took place once a week from 09.00 - 11.00 am, in order to separate male and female pupae. In our study, 24 replicates were conducted, with the total numbers ranging from 9000 to 25,000 (18,245 ± 4,973) male pupae that were separated and transported for sterilization by irradiation and then the later emerged adult males released at the field site.

### Quality of male and female pupae: weight, size and emergence time

After sex separation, *Wolbachia*-infected *Ae. aegypti* male and female pupae were introduced into plastic containers for further measurement and observation. They were then counted by using simple manual laboratory counting equipment. One thousand male or female pupae were separately placed in each plastic container half filled with water. A total number of 6 containers containing either male or female pupae were weighted and recorded in order to determine the weight of the pupae.

Three morphological characteristics, i.e. cephalothorax, abdomen and total length, were measured in 60 male and 60 female pupae [19, 20] in order to assess the difference in size. Each pupa was individually collected by using a dropper and was transferred into a small cup containing cold water (4 ± 2 °C) to make it immobilized. Then each pupa was placed on a glass slide and measured by the Olympus DP70 microscope (Tokyo, Olympus Corp.) using the DP Controller software (@2000 Olympus Optical Co., Ltd.).

In order to observe the developmental time, 1500 males and 1500 females of irradiated *Wolbachia*-infected pupae collected by using the dropper were transferred into a plastic bowl of 470 cm^3^ in volume and filled with 390 - 400 cm^3^ of water. The plastic bowl was then kept in a cage sized 30 cm x 30 cm x 30 cm and left in a screened insectary at a temperature of 27 ± 2 °C, 75 ± 2 % humidity and a photoperiod of L12:D12. A ten percent sucrose solution was provided inside the cage for emerged adult mosquitoes. Emerged pupae were observed from the onset of adult emergence (day 0) for five consecutive days (days 1, 2, 3, 4, 5). Three replicates of 500 male and 500 female pupae each were observed in this experiment.

### Sterilization of male pupae and screening for female contamination

Emerged *Wolbachia*-infected *Aedes aegypti* male pupae up to one day old were placed in plastic containers, each of 122.66 cm^3^ in volume (diameter 12.5 cm, height 14.5 cm) and with water of 62 cm^3^ in volume, prior to transportation to the radiation source. These plastic containers filled with male pupae were transported by air-conditioned car from the laboratory at Mahidol University Salaya Campus, Nakhon Pathom Province to the Thailand Institute of Nuclear Technology (Public Organization) (TINT), Nakhon Nayok Province, which is located 112 km. away. Using a Colbalt-60 (Gammar Chamber 5000, Board of Radiation and Isotope Technology (BRIT), DAE, Mumbai, India), an irradiation dose of 50 Gy or 70 Gy for 45 seconds was applied by a qualified staff at TINT. After irradiation, part of the irradiated male pupae were transported back to the laboratory for further experiments, while most of them were transported to the field station at the City Center of Chachoengsao Province. Then small plastic containers holding irradiated pupae were put in plastic release cages prior to adult emergence, and a 10 % sucrose solution was provided. After emergence, irradiated *Wolbachia*-infected males were double-checked for female contamination using a mouth aspirator to individually place male mosquitoes into the new plastic release cages. The number of mixed female mosquitoes from emerged pupae was recorded. The emerged irradiated *Wolbachia*-infected male mosquitoes, 1-3 days old, were then weekly released in the pilot trial to suppress *Ae. aegypti* mosquito vector populations at a village scale in Plaeng Yao District, Chachoengsao Province, Thailand. The number of released sterile male mosquitoes ranged from 9000 to 25,000 per week.

### Testing sterility of irradiated *Wolbachia*-infected male and female mosquitoes

Preliminary experiments were set up to test the sterility of *Ae. aegypti* male and female mosquitoes after being exposed to an irradiation dose of 50 Gy or 70 Gy. In addition, during the 24-week open field trial, each lot of irradiated males and females was tested for sterility by mating them with non-irradiated females and non-irradiated males, respectively. In the experiments, the irradiated *Wolbachia*-infected male and female mosquitoes were separately introduced into cages sized 30 cm × 30 cm × 30 cm, with a 10 % sucrose solution provided. The non-irradiated *Wolbachia*-infected females were then introduced into the cage with irradiated *Wolbachia*-infected males, while the non-irradiated *Wolbachia*-infected males were introduced into the cage with irradiated *Wolbachia*-infected females. The ratio of irradiated *Wolbachia*-infected male and non-irradiated *Wolbachia*-infected female mosquitoes was 1:1. The same ratio was applicable with irradiated *Wolbachia*-infected female and non-irradiated *Wolbachia*-infected male mosquitoes. The mosquitoes were freely mated in the cages for 2-3 days. The females were then blood-fed using the Hemotek blood-feeding system (Hemotek Ltd., UK). Each blood-feeding period lasted 1-2 hours and the Hemotek blood-feeding unit with new blood was re-introduced within 2-3 consecutive days. Blood-fed irradiated and non-irradiated *Wolbachia*-infected female mosquitoes were individually separated and placed in a plastic tube 7 cm^3^ in volume (diameter 3 cm, height 5.5 cm). Egg paper was placed over wet cotton inside each plastic tube for oviposition. After 3-4 days, the egg paper from each female mosquito was collected and the eggs were counted. Then it was dried and transferred into a glass container containing deionized water for hatching, as previously described. The number of hatched and un-hatched eggs from each individual female mosquito was recorded. The un-hatched eggs represented the sterility of the tested mosquitoes.

### Assessing survival and longevity of irradiated and non-irradiated *Wolbachia*-infected male and female mosquitoes

The emerged irradiated and non-irradiated *Wolbachia*-infected male and female mosquitoes were separately introduced into a cage sized 30 cm × 30 cm × 30 cm, with a 10 % sucrose solution provided. They were placed in the insectary at a temperature of 27 ± 2 °C, 75 ± 2 % humidity and a photoperiod of L12:D12. The number of dead male and female mosquitoes was daily observed and recorded. The dead mosquitoes were then removed from the cage.

### Statistical analysis

All statistical analyses were performed using SPSS 18.0 Mahidol University License (Chicago, SPSS Inc.). Weight, size, emergence, sex ratio of male and female pupae, sterility, and the longevity of irradiated *Wolbachia*-infected male and female mosquitoes were analyzed by means of one-way and two-way analyses of variance (ANOVA). Correlation between sex ratio and the number of released mosquitoes was analyzed by using Pearson's correlation.

## Results

### Weight, size and emergence time of male and female pupae

When comparing the weight of 1000 *Wolbachia*-infected *Ae. aegypti* male and female pupae, it was found that female pupae appeared to be heavier in weight, and this difference was statistically significant (*df *= 2*, F* = 74.940, *P* = 0.001). The weight of female pupae ranged from 5.12 - 5.70 mg (5.49 ± 0.32 mg), whereas those of the males ranged from 3.52 - 3.70 mg (3.69 ± 0.16 mg) (Table 1).

**Table 1:** Comparison of average weight of *Wolbachia*-infected *Aedes aegypti* male and female pupae after being sex separated by using larval-pupal glass separators (Model 5412, John W. Hock Company, Gainesville, FL, USA)

Sex	Rep.	*N* (Total)	Weight (mg) (Mean ± SD)	95% CI	*F*	*P*
Male	3	1000	3.69 ± 0.16	3.28–4.08	74.940	0.001*
Female	3	1000	5.49 ± 0.32	4.69–6.30		

*Significant difference at *P*< 0.05

When comparing the size of *Wolbachia*-infected *Ae. aegypti* male and female pupae, female pupae were significantly much bigger than male pupae in all parts, i.e., cephalothorax (female = 3.00 ± 0.11 mm *vs* male = 2.21 ± 0.43 mm, *t* = -12.948, *df* = 59, *P* = 0.000), abdomen (female = 3.13 ± 0.42 mm *vs* male = 2.40 ± 0.41 mm, *t* = -10.869, *df* = 59, *P* = 0.000) and body length (female = 5.46 ± 0.42 mm *vs* male = 4.08 ± 0.74 mm, *t* = -12.714, *df* = 59, *P* = 0.000) (Table 2). Difference in the pupae size was an important parameter that was beneficial to mechanical sex separation.

**Table 2:** Average size of *Wolbachia*-infected *Aedes aegypti* male and female pupae classified by cephalothorax, abdomen, and body length after being sex separated by using larval-pupal glass separators (Model 5412, John W. Hock Company, Gainesville, FL, USA)

Morphology	Rep.	Size (mm.) (Mean ± SD)	95% CI	*t*	*df*	*P*
Cephalothorax						
Male	60	2.21 ± 0.43	-0.91– -0.67	-12.948	59	0.0001*
Female	60	3.00 ± 0.11				
Abdomen						
Male	60	2.40 ± 0.41	-0.86– -0.59	-10.869	59	0.0001*
Female	60	3.13 ± 0.42				
Body length						
Male	60	4.08 ± 0.74	-1.60– -1.17	-12.714	59	0.0001*
Female	60	5.46 ± 0.42				

*Significant difference at *P* < 0.05

When comparing emergence time between irradiated *Wolbachia*-infected male and female pupae, our results demonstrated that most male and female pupae emerged into adult mosquitoes on the second day after reaching the pupal stage, accounting for 74.60 % and 62.05 % respectively. A significant difference in emergence time was observed between male and female pupae (*df = 3, F*_male_ = 735.025, *P* = 0.000; *df* = *3, F*_female_ = 232.464, *P* = 0.000) (Table 3). However, no significant difference was observed between the number of male and female pupae that emerged into adult mosquitoes (*t* = -0.15, *df* = 11, *P* = 0.989). It was observed that more than 98 % of male and female pupae emerged into adults, which means that the irradiation dose of 70 Gy did not have negative effect on their emergence.

**Table 3:** Average number of emerged *Wolbachia*-infected *Aedes aegypti* male and female pupae after being irradiated at 70 Gy

Sex	Rep.	*N* (Total no.)	Day	Average emerged mosquitoes (Mean ± SD)	Average non-emerged mosquitoes	% emerged mosquitoes	95% CI	*F*	*P*
Male	3	1500	1	41. 67 ± 12.86	0.00 ± 0.00	8.33 ± 2.57	1.95–14.72	735.025	0.00*
			2	369.00 ± 14.73	0.00 ± 0.00	73.80 ± 2.95	66.48–81.12		
			3	80.67 ± 7.77	0.00 ± 0.00	16.13 ± 1.55	12.27–19.99		
			4	3.33 ± 3.21	5.33 ± 4.04	0.67 ± 0.64	-0.93–2.26		
Female	3	1500	1	13.33 ± 7.09	0.00 ± 0.00	2.67 ± 1.42	-0.86–6.19	232.464	0.00*
			2	306.33 ± 23.44	0.00 ± 0.00	61.27 ± 4.69	49.62–72.91		
			3	168.00 ± 21.28	0.00 ± 0.00	33.60 ± 4.26	23.03–44.17		
			4	6.00 ± 1.73	6.33 ± 1.15	1.20 ± 0.35	0.34–2.06		

*Significant difference at *P* < 0.05

### Percentage of female contamination during pilot field release

**Fig. 1:**
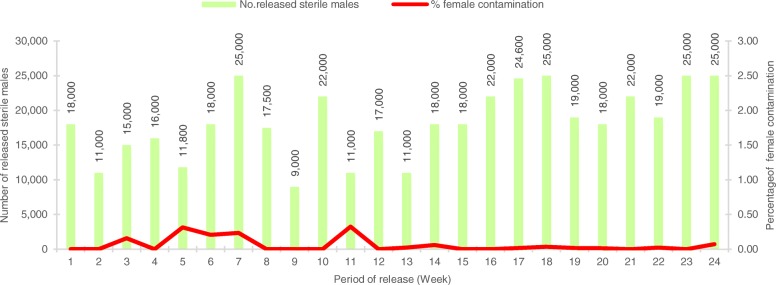
Percentage of female contamination after mechanically sex separated to obtain sterile males for use in the pilot open field release, in order to suppress natural *Aedes aegypti* populations

During the twenty-four weeks of the pilot field trial and the releases of sterile *Ae. aegypti* males at the selected study site in Plaeng Yao District, Chachoengsao Province, each lot of sterile male pupae ranging from 1282 to 23,481 (mean ± SD = 5107.29 ± 4,509.59) was inspected for female contamination. In total, 122,575 sterile male pupae were inspected for female contamination and the data are presented in Fig. 1 and Table 4. Results indicate that a total number of 60 females, ranging from 0 to 17, were found mixing with males during the mechanical sex separation process (mean ± SD = 2.50 ± 4.17) (Table 4), accounting for 0.06 ± 0.10 % female contamination. Remarkably, when 1282 to 5,000 sterile males were inspected during the first twelve weeks of the intervention, the female contamination was 0.10 ± 0.13 % (2.40 ± 3.42). However, there were lower numbers of female contamination during the second twelve weeks of intervention, i.e. 0.02 ± 0.02 % (2.67 ± 5.43), even though samples containing more than 5000 sterile males were inspected, and a statistically significant difference was observed between the two groups of intervention (*t* = 2.317, *df* = 11, *P* = 0.041) (Table 4). It is worth noting that when the largest sample was applied for sex separation (23,481 male pupae), the female contamination was 0.07 %. When compared between the first and second 12-week periods, it was noticed that, there was a fluctuation in the percentage of female contamination during the first twelve weeks of release, but this was relatively small, accounting for 0.02 – 0.32 %. This percentage of female contamination was remarkably reduced in the second twelve weeks of intervention. In conclusion, at least 99 % of sterile males were purely separated from females, demonstrating high efficiency in the manual sex separation process during this pilot intervention.

**Table 4:** Percentage of female contamination after mechanical sex separation, comparing the first and the second twelve weeks of intervention

Replicate	No. sampling sterile males	% female contamination (Mean ± SD)	95% CI	*df*	*t*	*P*
12	38,253	0.11 ± 0.13	0.004–0.169	11	2.317	0.041*
12	84,322	0.02 ± 0.02				

*significant difference at *P * < 0.05

### Sterility of irradiated *Wolbachia*-infected male and female mosquitoes

A preliminary study showed that an irradiation dose of 50 Gy was sufficient to induce complete sterility in *Ae. aegypti* females but not in males (Table 5). *Wolbachia*-infected males irradiated (♂ir-w) at 50 Gy could still produce viable eggs when mated with non-irradiated *Wolbachia*-infected females (♀nr-w). The average percentage of eggs hatched into the first-instar larvae was 8 %, while the egg hatch rate was zero when irradiated *Wolbachia*-infected females (♀ir-w) mated with non-irradiated *Wolbachia*-infected males (♂nr-w). No eggs were hatched when *Wolbachia*-infected males and females were irradiated (♂ir-w & ♀ir-w) at 70 Gy and then were mated with non-irradiated *Wolbachia*-infected females and males (♀nr-w & ♂nr-w) respectively.

**Table 5:** Sterility of irradiated *Wolbachia*-infected male and female *Aedes aegypti* mosquitoes after being exposed to the irradiation dosages of 50 Gy or 70 Gy

Experiment	No. of females (F0)	No. of egg-laid females (F0)	Total no. of eggs	Eggs/female	No. of hatched eggs	Egg hatch rate
Radiation dosage						
50 Gy						
♂ ir-w x × ♀ nr-w	27	23	1,021	44.39	80	0.08
♂ nr-w × ♀ ir-w	27	0	0	0	0	0
70 Gy						
♂ ir-w × ♀ nr-w	18	10	404	40.40	0	0
♂ nr-w × ♀ ir-w	27	0	0	0	0	0

**Fig. 2:**
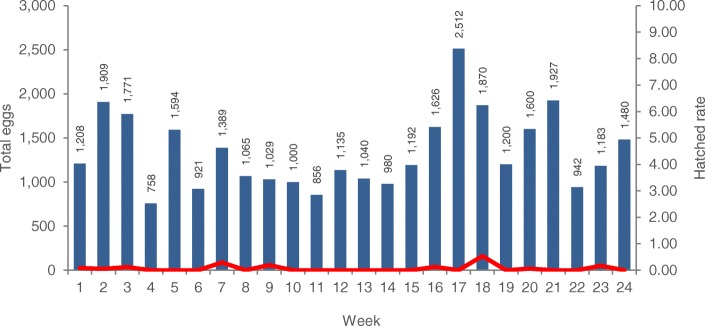
Total number of eggs and egg hatch rate from mating between irradiated *Wolbachia*-infected males and non-irradiated *Wolbachia*-infected females of *Aedes aegypti* mosquitoes

Results of mating tests performed during the 24-week open field trial between irradiated and non-irradiated *Wolbachia*-infected *Ae. aegypti* male and female mosquitoes are shown in Fig. 2 and Table 6. When irradiated *Wolbachia*-infected males mated with non-irradiated *Wolbachia*-infected female mosquitoes, the females could still lay eggs (mean _total eggs_ = 1,341.13 ± 431.61), but the number of hatched eggs was quite low (mean _hatched eggs_ = 1.04 ± 2.18) (Fig. 2, Table 6), which demonstrates that the irradiated *Wolbachia*-infected males were highly sterile and that their sterility could provoke nearly complete sterility through reduction of the egg hatch rate in the next generation (mean _hatch rate_ = 0.07 ± 0.13). Irradiated *Wolbachia*-infected female mosquitoes seemed to be more sensitive to 50 Gy and 70 Gy irradiation doses, since they lost their ability to lay eggs (mean _total eggs_ = 0.00 ± 0.00) or they were completely sterile when mated with non-irradiated *Wolbachia*-infected males (mean _hatch rate_ = 0.00± 0.00) (Tables 5 and 6).

**Table 6:** Analysis of variance of total number of eggs, hatched eggs and egg hatch rate between irradiated *Wolbachia*-infected (♂ir-w) males and non-irradiated *Wolbachia*-infected (♀nr-w) females vs non-irradiated *Wolbachia*-infected (♂nr-w) males and irradiated *Wolbachia*-infected (♀ir-w) females of *Aedes aegypti* mosquitoes

Mating pair	Rep.	*N*	Mean ± SD	95% CI	*t*	*df*	*P*
Total eggs							
♂ir-w x ♀nr-w	24	1110	1341.13 ± 431.61	1158.87–1523.38	15.22	23	0.0001*
♂nr-w x ♀ir-w	24	1105	0.00 ± 0.00				
Hatched eggs							
♂ir-w x ♀nr-w	24	1110	1.04 ± 2.18	0.12–1.96	2.35	23	0.028*
♂nr-w x ♀ir-w	24	1105	0.00 ± 0.00				
Egg hatch rate							
♂ir-w x ♀nr-w	24	1110	0.07 ± 0.13	0.01–0.12	2.60	23	0.016*
♂nr-w x ♀ir-w	24	1105	0.00 ± 0.00				

*Significant difference at *P* < 0.05

When comparing the crosses between non-irradiated *Wolbachia*-infected females mated with irradiated *Wolbachia*-infected males (nr-w female × ir-w male) and irradiated *Wolbachia*-infected females mated with non-irradiated *Wolbachia*-infected males (ir-w female × nr-w male), there were statistically significant differences in the total number of eggs (*t* = 15.22, *df* = 23, *P* = 0.000), the hatched eggs (*t* = 2.35, *df* = 23, *P* = 0.028) and the egg hatch rate (*t* = 2.60, *df* = 23, *P* = 0.016) (Table 6). In conclusion, an irradiation dose of 70 Gy induces high to nearly complete sterility in male mosquitoes. Non-irradiated *Wolbachia*-infected females that mated with irradiated *Wolbachia*-infected males could still lay eggs (mean _total eggs_ = 1,341.13 ± 431.61 eggs), but the number of hatched eggs was quite low (mean _hatched eggs_ = 1.04 ± 2.18), which demonstrated that the irradiated *Wolbachia*-infected male mosquitoes were highly sterile. Contrary to males, irradiated *Wolbachia*-infected females with a dose of 70 Gy either lost their ability to lay eggs (mean _total eggs_ = 0.00 ± 0.00 eggs) after mating with non-irradiated *Wolbachia*-infected males or were completely sterile (mean _hatch rate_ = 0.00± 0.00) (Table 6). Therefore, since the males and females used in the crosses were both *Wolbachia*-infected, sterility should be induced by irradiation, with a dose of 70 Gy being the optimum one for *Wolbachia*-infected *Ae. aegypti* male mosquitoes, as it could make them fully sterile. Moreover, in the case where irradiated *Wolbachia*-infected females were accidentally released, these females could not reproduce because they were fully sterile due to the effect of irradiation.

### Longevity and survival rate of irradiated and non-irradiated *Wolbachia*-infected males and females following sex separation

Overall, adult longevity in irradiated *Wolbachia*-infected *Ae. aegypti* mosquitoes varied from 3 to 44 days, while for non-irradiated *Wolbachia*-infected *Ae. aegypti*, it was from 2 to 71 days. The differences in longevity and survival rate between male and female mosquitoes were mostly observed from 12 to 35 days.

**Fig. 3:**
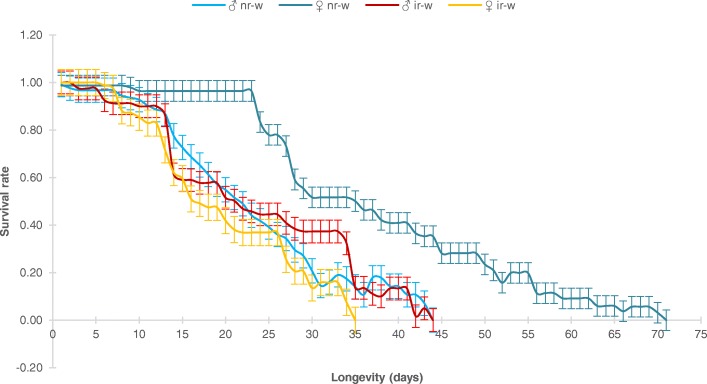
Mean longevity and survival rate of non-irradiated and irradiated *Wolbachia*-infected *Aedes aegypti* male and female mosquitoes, after being sex-separated by using larval-pupal glass separators

For irradiated *Wolbachia*-infected mosquitoes, males seemed to live longer and had a higher survival rate when compared to females (longevity: ♂ir-w = 22.14 ± 11.44 days *vs* ♀ir-w = 18.47 ± 9.81 days; survival rate: ♂ir-w = 0.63 ± 0.26 *vs* ♀ir-w = 0.55 ± 0.32), and the differences were statistically significant (*t* = 5.962, *df* = 34, *P* = 0.000) (Fig. 3, Table 7). On the contrary, the non-irradiated *Wolbachia*-infected females lived longer and had a higher survival rate, when compared to the non-irradiated *Wolbachia*-infected males (longevity: ♀nr-w = 29.64 ± 1.03 days *vs* ♂nr-w = 23.31 ± 0.91 days; survival rate: ♀nr-w = 0.76 ± 0.25 *vs* ♂nr-w = 0.51 ± 0.34), and the differences were statistically significant (*t* = -10.687, *df* = 43, *P* = 0.000).

**Table 7:** Analysis of variance of longevity and survival rate between irradiated *Wolbachia*-infected (ir-w) and non-irradiated *Wolbachia*-infected (nr-w) *Aedes aegypti* male and female mosquitoes, after being sex-separated by using larval-pupal glass separators

Experiment	*N*	Longevity (day) (Mean ± SD)	Survival rate	95% CI	*t*	*df*	*P*
♂ ir-w	120	21.11 ± 10.14	0.63 ± 0.26	0.06–0.11	5.962	34	0.0001*
♀ ir-w	120	18.47 ± 9.81	0.55 ± 0.32				
♂ nr-w	120	23.31 ± 0.91	0.51 ± 0.34	-0.29– -0.20	-10.687	43	0.0001*
♀ nr-w	120	29.64 ± 1.03	0.76 ± 0.25				
♂ ir-w	120	23.77 ± 12.11	0.52 ± 0.32	-0.02–0.04	0.795	43	0.431
♂ nr-w	120	23.31 ± 0.91	0.51 ± 0.34				
♀ ir-w	120	18.47 ± 9.81	0.55 ± 0.32	-0.37– -0.23	-8.738	34	0.0001*
♀ nr-w	120	25.44 ± 1.07	0.85 ± 0.19				

*Significant difference at *P* < 0

However, when comparing either males or females between irradiated and non-irradiated, we found no difference in longevity and survival rate between the irradiated *Wolbachia*-infected males and the non-irradiated *Wolbachia*-infected males (longevity: ♂ir-w = 23.77 ± 12.11 days *vs* ♂nr-w = 23.31 ± 0.91 days; survival rate: ♂ir-w = 0.52 ± 0.32 *vs* ♂nr-w = 0.51 ± 0.34, *t* = 0.795, *df* = 43, *P* = 0.431). On the contrary, the irradiated *Wolbachia*-infected females have a much shorter lifespan and low survival rate when compared to the non-irradiated *Wolbachia*-infected females (longevity: ♀ir-w = 18.47 ± 9.81 days *vs* ♀nr-w = 25.44 ± 1.07 days; survival rate: ♀ir-w = 0.55 ± 0.32 *vs* ♀nr-w = 0.85 ± 0.19), and the differences were statistically significant (*t* = -8.738, *df* =34, *P* = 0.000).

## Discussion

Size difference in male and female pupae was the basis for sex separation, especially for mass production of sterile males to be used in the SIT programmes. Various factors such as larval density, diet, temperature and others affect pupae size, and a standardized rearing condition was required in order to effectively separate males from females [15, 19]. In our study, female pupae appeared to be significantly larger than males and could easily be separated by mechanical tools, indicating our appropriate rearing condition. Importance of male size has previously been highlighted in the mating success, larger males having a greater mating capacity than smaller males [21–24].

Sex separation at the pupal stage was more convenient and practical when compared to adults [25]. Moreover, late pupae were more tolerant to the irradiation process than early ones, in terms of an effect on adult emergence and mortality. In our study, the larval-pupal glass separators were used in sex separation, and a high survival rate was observed. More than 62 % and 74 % of female and male pupae respectively emerged on the second day. Therefore, it was more practical and recommended to sex separate them on the second day of pupation, when there was still a high percentage of males, as indicated in Medici et al. (2011) [26]. Some studies reported using metal sieves to separate male from female pupae with a high purity, but only 15 - 25% of males were recovered [15].

In the past sterile male release programmes, only less than 5 % of female contamination was acceptable, but currently this is considered as unacceptable [12]. In this study, at least 99 % of male pupae were successfully separated, and more than 98 % of males recovered, after being irradiated. Therefore, sex separation at the pupal stage by using adjustable glass plates could be an appropriate method to use for small-scale pilot trials. However, care should be taken in terms of maintaining harmonized adjustment at the early stage of sex separation. Skill of the personnel working with manual sex separation using larval-pupal glass separators was important to obtain a high percentage of male pupae and minimal female contamination. Our pilot intervention showed a fluctuation in the percentage of female contamination during the first twelve weeks, but not in the second period when the personnel were more skillful and more familiar with the technique.

Different mosquito species require different irradiation dose to achieve complete sterility. In our experiments, a difference in susceptibility to irradiation was observed between the male and female *Ae. aegypti* mosquitoes, as reported in other insects [3, 4]. Females were more susceptible to irradiation than males, being completely sterile at a lower dose. When no perfect sex separation method is available, female mosquitoes could accidentally be released together with males. Therefore, it is necessary to consider an irradiation dose that could fully sterilize both male and female mosquitoes, in order to eliminate the risk of releasing fertile mosquitoes. In our case, any released females not only would be sterile but also would exhibit reduced risk of pathogen transmission, since they were infected with *Wolbachia* [27].

In a system of SIT/IIT, the sterility of released males would be due to both *Wolbachia* and low-dose irradiation, while the *Wolbachia*-infected female sterility would only be caused by irradiation. In our preliminary study, irradiation doses at both 50 Gy and 70 Gy could induce complete sterility in *Wolbachia*-infected *Ae. aegypti* females; but at 50 Gy, only female but not male mosquitoes were completely sterile. Non-irradiated *Wolbachia*-infected females that mate with irradiated *Wolbachia*-infected males still produced hatched eggs, although at very low egg hatch rate of 0.08. The sterility induced in these females was due to irradiation, as both males and females were *Wolbachia*-infected and our previous work indicated incomplete CI when non-irradiated *Wolbachia*-uninfected females were mated with non-irradiated *Wolbachia*-infected males [18]. But for the irradiated *Wolbachia*-infected females, this dosage induced complete sterility and no egg production was observed. For the quality control test of all 24 lots of mosquitoes which were irradiated at 70 Gy, we found a very low egg hatch rate of 0.07 of all crosses between irradiated *Wolbachia*-infected males and non-radiated *Wolbachia*-infected females. Hence, irradiation at 70 Gy was considered the practical dose to induce sterility in *Wolbachia*-infected *Ae. aegypti* male mosquitoes for being released in our pilot field suppression trial. Based on the above, the combined SIT/IIT offers a safe and biosecure approach for population suppression programmes against *Ae. aegypti* similar to the one recently developed and applied against *Aedes albopictus* [8–10].

In terms of longevity, female mosquitoes were reported to live up to 90 days [28] or, in some cases, up to 150 days [29, 30]. Our results showed a significant impact of irradiation on female longevity and survival rate. The longevity of irradiated *Wolbachia*-infected *Ae. aegypti* females was reduced to nearly 18 days on average. In the case of males, our results showed almost no effect on longevity and survival rate, since no significant differences were observed between irradiated and non-irradiated *Wolbachia*-infected *Ae. aegypti* males.

In our study, we found that irradiated *Wolbachia*-infected males lived slightly longer than irradiated *Wolbachia*-infected females. However, our results contradict other studies [28, 31] that reported longer lifespans among females than males, whether both sexes were reared separately or together. The difference in longevity between males and females demonstrated in our study is related to the fact that females are more radiosensitive than males. Radiation has been shown to decrease adult life span, including its subsequent generations [21, 32]. Moreover, changes in hatchability, followed by adult emergence and longevity, were more prominently observed with increasing irradiation dose [32]. At the pupal stage, irradiation can negatively affect adult emergence and consequently survival rates [8]. In this study, irradiated *Wolbachia*-infected females exhibited higher mortality when compared to irradiated *Wolbachia*-infected males when they were exposed at the same irradiation dose. However, irradiated *Wolbachia-*infected females in our study were only fed with a sucrose solution and no blood meals were provided. This could be one of the important parameters to explain for the shorter lifespan of these females. Other studies have shown that *Ae. aegypti* females fed only on a sucrose solution had a shorter lifespan that those fed with either blood alone or both blood and sugar [33–35]. This observation was most likely due to a depletion of protein reserves [33].

## Conclusions

In conclusion, application of SIT requires various components, including a mass-rearing process that consists of many important parameters, such as diet, rearing conditions, and importantly sex separation. Our study provides useful information in terms of the practical application of a mechanical sex separation method to obtain only males for further sterilization and open small-scale field release. However, this approach would not be effective for a very large scale application. There is certainly an urgent need for further research to develop novel, efficient and cost-effective sex separation techniques to support large scale SIT applications [13, 36]. We also demonstrated the quality of male and female mosquitoes, in terms of survival and longevity after sex separation and sterilization, which would be beneficial for planning a field release in order to suppress natural populations of *Ae. aegypti* mosquitoes. In our studies, we found no significant difference between non-radiated and irradiated males in term of survival and longevity while irradiated females had shorter life span. In addition, both irradiated males and females were completely sterile when they were irradiated at 70 Gy. The overall quality including the male mating competitiveness of the released irradiated *Wolbachia*-infected *Ae. aegypti* mosquitoes needs to be further investigated particularly because this is a critical factor for the successful field implementation of the combined SIT/IIT approach.
